# Intermittent low‐intensity contractions cause a dissociation between maximal motor unit firing rates and changes in torque and contractile speed

**DOI:** 10.1113/EP093505

**Published:** 2026-08-04

**Authors:** Alexander M. Zero, Raaj A. Dudani, Jacob Fanous, Charles L. Rice

**Affiliations:** ^1^ School of Kinesiology Faculty of Health Sciences The University of Western Ontario London Ontario Canada; ^2^ Department of Human Health Sciences College of Biological Sciences University of Guelph Guelph Ontario Canada; ^3^ Section of Anatomy Department of Surgery University of Texas Southwestern Medical Center Dallas Texas USA; ^4^ Department of Anatomy and Cell Biology Schulich School of Medicine & Dentistry The University of Western Ontario London Ontario Canada

**Keywords:** contractile properties, discharge rate, electrical stimulation, neuromuscular fatigue, peripheral feedback, rate coding

## Abstract

The purpose was to test: (1) whether the decline in maximal motor unit (MU) firing rate is an invariable response when maximal voluntary contraction (MVC) torque is reduced following a submaximal muscle fatiguing task; and (2) whether fatigue‐induced slowing of contractile speed and firing rate decline are co‐dependent. Ten participants (three females) underwent a protocol (duty cycle, 0.6 s) of knee extensions at 30% MVC for 30 min, which has been shown previously to reduce MVC torque with negligible muscle metabolic disturbances. Electrically evoked quadriceps torque (1, 50 and 100 Hz), MVC torque and maximal firing rates were assessed intermittently throughout the protocol. Intramuscular microelectrodes were inserted into the vastus lateralis and medialis to record MU firing rates (*n* = 1557). By 30 min, MVC and electrically evoked quadriceps torques declined (30%–50%, all *P* ≤ 0.02); however, measures of contractile speed increased (∼30%, all *P* < 0.001). Grouped mean maximal firing rates were not significantly changed (24.1 ± 8.9 Hz) from pre‐task (24.7 ± 9.2 Hz, *P* = 0.16). Therefore, reductions in maximal torque generation, contractile slowing and decreased maximal MU firing rates are not invariable correlates of a fatiguing task. Instead, reduced MVC torque can be dissociated from the usual slowing of maximal firing rates and contractile speed when fatigue is induced with, presumably, negligible metabolic disturbances. The constancy of maximal firing rates despite large reductions in MVC torque supports that firing rate moderation with fatiguing activation is probably attributable to reflexive inhibition from muscle metabolic alterations and thus not directly related to changes in contractile speed.

## Abstract (highlights)

- **What is the central question of this study?**
  What are the responses of maximal motor unit (MU) firing rates and muscle contractile speed when maximal torque‐generating capacity is decreased by intermittent low‐intensity contractions?
- **What is the main finding and its importance?**
  Following submaximal fatiguing activation, with presumably negligible metabolic disturbances, maximal MU rates were unchanged despite large reductions in maximal torque and increases in contractile speed. The unchanged maximal firing rates despite large reductions in maximal torque indicate that MU rate reductions with fatiguing activation are probably attributable to reflexive feedback inhibition from changes in muscle metabolites and are not directly related to changes in contractile speed.

## 1 INTRODUCTION

Prolonged muscle activation leads to an objective reduction in the contractile response (e.g., torque), which is referred to as muscle fatigue (Allen et al., 2008; Bigland‐Ritchie et al., 1995; Taylor et al., 2016). During a sustained (e.g., 60 s) maximal voluntary isometric contraction (MVC), torque and motor unit (MU) firing rates can decline upwards of 50%, and electrically evoked muscle relaxation rates can slow by ∼70% (Bigland‐Ritchie et al., 1983a; Bigland‐Ritchie & Woods, 1984; Grimby et al., 1981; Zero et al., 2023). Therefore, in addition to reduced torque generation, contractile slowing is a hallmark feature associated with sustained high‐intensity fatiguing tasks (Bigland‐Ritchie & Woods, 1984; Davis & Davis, 1932; Edwards et al., 1975).

It was originally hypothesised that the fatigue‐induced reduction in MU firing rates was preventive, rather than a contributor to force (torque) loss (i.e., muscle wisdom; Bigland‐Ritchie et al., 1979; cf. Fuglevand & Keen, 2003; Jones et al., 1979; Marsden et al., 1983). This hypothesis states that the lowered MU firing rates during sustained MVCs would maintain adequate muscle excitation as contractile speed slows with fatigue (for a review, see Zero & Rice, 2025). Indeed, this has been supported during sustained high‐intensity isometric activation (Bigland‐Ritchie et al., 1979; Dudani et al., 2025; Jones et al., 1979; Marsden et al., 1983). However, despite this functionally sensible central–peripheral matching it is not clear whether firing rate reduction and contractile slowing are a co‐dependent phenomenon.

To test whether contractile slowing modifies MU firing rate independently, Bigland‐Ritchie and colleagues performed separate experiments in which contractile speed was altered through modifications in joint angle (muscle length) and muscle temperature (Bigland‐Ritchie et al., 1992a, 1992b). Despite the expected changes in contractile speed, maximal firing rates were unaffected in both experiments (Bigland‐Ritchie et al., 1992a, 1992b). Therefore, in the absence of muscle fatigue, altering contractile speed did not independently modify firing rates. However, that dissociation has not been investigated during the stress of fatiguing contractions, in which various compensatory factors within the neuromuscular system, presumably to mitigate torque decline, have been evaluated.

In separate experiments by the same group, it was concluded that the metabolites that accumulate in the active muscle during sustained MVCs (e.g., H^+^, K^+^, P_i_) initiate a reflexive inhibition that lowers maximal MU firing rates. Experiments demonstrated that when ischaemia was maintained with a blood pressure cuff following fatiguing elbow‐flexion MVCs, maximal firing rates of the biceps brachii remained reduced. Subsequently, when the cuff was removed and normal perfusion restored, firing rates recovered to ∼95% by 3 min of rest (Bigland‐Ritchie et al., 1986). These observations were also replicated in the knee extensors using similar protocols (Woods et al., 1987). Therefore, the interpretation was that in this task the fatigue‐induced changes in the muscle, which are likely to stimulate mechanical and chemical sensitive afferents (i.e., group III/IV), appear to regulate MU firing rates from peripheral and muscle feedback.

During fatiguing contractions, slowed relaxation is often attributed to accumulation of muscle metabolites (Cady, Elshove et al., 1989; Dawson et al., 1980; Edwards et al., 1975). Therefore, as indicated above, it is likely not to be the contractile slowing per se that reduces firing rates in this task, but rather the fatigue‐induced changes in the muscle (e.g., H^+^ accumulation) that slows both relaxation rates and maximal MU firing rates. Thus, this hypothesis predicts that during fatigue (defined here as torque loss following contractile activation) in which no or minimal metabolites accumulate within the muscle, the maximal MU firing rates would not decline; however, this has not been tested systematically. This is a logical and important next step in resolving whether and how changes in the peripheral muscle contractility impact neural output through the final common pathway of MU activation.

Therefore, the purpose was to test: (1) whether the decline in maximal MU firing rates is an invariable response when MVC torque is impaired; and (2) whether slowed contractile speed and the decline in maximal firing rates are co‐dependent during fatiguing contractions. To address this, we repeated an intermittent submaximal fatiguing protocol that reduced MVC torque by ∼40%, but, notably, negligible metabolic disturbances occurred (Vøllestad et al., 1988; see section 2). We hypothesized that: (1) despite significant reductions in MVC torque, maximal MU firing rates will not decline throughout the fatiguing task; and (2) muscle contractile speed will not demonstrate the usual fatigue‐induced contractile slowing consistently reported during high‐intensity contractions.

## 2 MATERIALS AND METHODS

### 2.1 Ethical approval

The research described was approved by the local university review board for health science research involving human participation (no. 107505). Oral and written consent was obtained from all participants before any experimental testing. The study conformed to the standards set by the *Declaration of Helsinki*, except for registration in a database.

### 2.2 Participants

Seven males (25.7 ± 2.8 years, 179.1 ± 9.1 cm, 76.95 ± 8.2 kg) and three females (22.7 ± 2.1 years, 164.5 ± 1.8 cm, 60.9 ± 5.2 kg) volunteered for this study. Verbal screening indicated that participants were free from neuromuscular and metabolic disorders. Participants were instructed to avoid any strenuous or unaccustomed exercise for 48 h prior to the testing sessions. Participants were not currently involved in any regimented exercise programme but were considered recreationally active. The sample size (*n* = 10) was selected to reflect the original study from which the current fatiguing task was closely replicated (see section 2.6 Fatiguing protocol, *n* = 13; Vøllestad et al., 1988) and supported by a sample size calculation (*n* = 9 for 0.8 power) based on the data estimated from Vøllestad et al. (1988) (see their Figure 2) using the package *WebPower* in R (v.4.1.2).

### 2.3 Experimental arrangement

Involuntary knee extension was evoked electrically using two percutaneous stimulation electrodes. The electrodes were custom made, consisting of layered aluminum foil wrapped in conductive gel and salt water‐soaked cloth (Roos et al., 1999). Participants performed knee extension and thigh adduction such that the boarders of the quadriceps and adductor muscles could be delineated visually and with palpation. The electrodes were modified and shaped to fit each participant (12–20 cm × 6–15 cm) and were then secured transversely to the proximal and distal portions of the right thigh over the quadriceps. The proximal electrode was 5–10 cm distal of the greater trochanter, and the second electrode was placed 5–15 cm distal to the proximal one (Zero et al., 2024).

The right knee extensors were tested in a Cybex dynamometer (Cybex, Humac Norm dynamometers, Computer Sports Medicine Inc). Participants were seated upright. A harness seat belt was used to stabilize the hip and shoulders firmly, and a non‐elastic strap was secured across the right thigh to minimize extraneous movements. The right knee joint was fixed at 85° and the hip joint at 110°. The knee joint was aligned with the axis of rotation of the dynamometer. Isometric torque recorded from the dynamometer was converted from analog to digital (Power 1401, Cambridge Electronic Design, Cambridge, UK) and sampled at 1000 Hz (Spike2, Cambridge Electronic Design), and torque traces were displayed in real time from a 61 cm monitor placed 1 m away from the participant.

### 2.4 Electrical stimulation

All electrical stimulation was conducted with square‐wave pulses of 500 µs, with the stimulator (DS7AH, Digitimer Ltd, Welwyn Garden City, UK) set at maximal voltage (400 V). Using a single electrical pulse, stimulation intensity was increased in incremental steps (∼25 mA) until muscle twitch torque amplitude did not increase, despite a continued increase in current. To ensure that maximal stimulation was maintained throughout the session, the current was increased by a further ∼15% (170–485 mA). This maximal current was used for all subsequent contractions using a single pulse (i.e., muscle twitch) and doublet (100 Hz) stimulation. A lower current (60–160 mA) was used for 50 Hz stimulation, which lasted 1 s (see sections 2.7 Day 1 and 2.8 Days 2 and 3).

### 2.5 Electromyography

Using the same techniques as those from the original and many subsequent studies of fatigued‐related changes in MU firing rates, single MU activity was recorded with custom‐made insulated tungsten microelectrodes (250 µm shank diameter, 45 mm in length and <1 µm tip diameter; FHC Inc., Bowdoin, ME, USA; Bellemare et al., 1983; Bigland‐Ritchie et al., 1983b, 1986; Dalton et al., 2010; Zero et al., 2025). This approach provides a large sample of MUs to create a ‘population’ mean and is especially suitable for achieving unit discrimination from MVC. Motor units are not individually tracked from contraction to contraction, but an overall mean is acquired from the recording of many units from various regions and depths of the muscles.

Prior to electrode insertion, the skin over the right vastus lateralis and medialis was cleansed with 70% ethanol. Two experimenters independently inserted and manipulated one sterile microelectrode each into the muscle belly of the vastus lateralis (separated by ∼15 cm), and one experimenter inserted and manipulated one microelectrode into the vastus medialis. All intramuscular signals were pre‐amplified (×100), filtered between 10 Hz and 10 kHz (Neurolog, NL844, Digitimer) and sampled at 20 kHz (Spike2, Cambridge Electronic Design) but were connected to independent channels. All were referenced to a surface electrode (GE HealthCare, ECG electrodes, 1 cm × 1 cm) placed over the patellar ligament, and a surface earth electrode was placed on the patella.

The two experimenters recording from the vastus lateralis had independent audio and visual feedback, which aided in recording quality as the electrodes were slowly advanced (∼1–5 mm per contraction) and manipulated during and between contractions to record from varying muscle depths and locations within the muscle (Bigland‐Ritchie et al., 1992a). Electrode movement was autonomous for each experimenter and was based on independent real‐time visual and audio feedback, whereas the experimenter recording from the vastus medialis was provided with visual feedback. Throughout the experiment, the microelectrodes were removed and reinserted into new locations along the muscle belly about five times per testing session to record a broad MU sample (Dalton et al., 2010) and to reduce the possibility of sampling from the same MU (Rich et al., 1998). With this technique and protocol, recruitment thresholds and the individual activation history of each MU cannot be determined. However, a large population profile can be obtained to represent well the overall behaviour of the motoneuron pool innervating the muscles.

Surface electromyography (sEMG) was recorded over the muscle belly of the vastus lateralis muscle using self‐adhering electrodes (GE Healthcare, ECG electrodes) in a bipolar arrangement. The active electrode was placed midway along the length of the muscle belly, and the reference electrode was placed 3–4 cm distally. The earth electrode was placed on the patella. The sEMG signals were pre‐amplified (×100), filtered between 10 Hz and 10 kHz (Neurolog, NL844, Digitimer, Welwyn Garden City, UK), with a 2500 Hz sampling rate (Spike2, Cambridge Electronic Design).

### 2.6 Fatiguing protocol

Participants performed the same fatiguing protocol over three visits (separated by 5.9 ± 2.3 days). The protocol was closely replicated from a prior report by Vøllestad et al. (1988) that showed negligible changes in fatigue‐induced muscle metabolites from biopsies taken throughout the fatiguing task despite a reduction in MVC torque by ∼40%. Specifically, intramuscular concentrations of glycogen, lactate, creatine phosphate and ATP did not change significantly (Vøllestad et al., 1988). Presumably, these substrates are replenished when blood flow is transiently restored during the brief rest periods between contractions and, furthermore, the low contraction intensity limits contraction‐induced blood flow restriction (Barcroft & Millen, 1939; Vøllestad et al., 1988). With a few minor practical modifications outlined below, the protocol consisted of intermittent (duty cycle, 0.6 s) knee extensions at 30% MVC for 30 min (Figure 1a). In the original experiments (Vøllestad et al., 1988), 50 Hz stimulation and brief MVCs were performed initially at 1 min, then at 5 min intervals until ‘exhaustion’ (upwards of 50 min). To mimic the original biopsy time points of 5, 15 and 30 min and to limit the fatiguing influences of the brief MVCs and 50 Hz, our test sequences (see sections 2.7 Day 1 and 2.8 Days 2 and 3) were performed only at 1, 5, 15 and 30 min. A schematic diagram of the protocol is presented in Figure 1a, b. Metabolic measures were not made in the present study, but essentially, we repeated the protocol from the prior study and in support the changes in contractile function here were very similar to those reported in the prior study (see section 3 RESULTS; Vøllestad et al., 1988), thus providing further confidence that metabolic influences were comparable to those of the original study. Indeed, there are several examples in which force loss can be dissociated from metabolic disturbances (Cady, Jones et al., 1989; Saugen et al., 1997). Furthermore, the expected minimal metabolic disturbance during this protocol is markedly different from those expected during sustained high‐intensity contractions, in which reductions in maximal MU firing rates have commonly been observed (cf. Kent‐Braun, 1999; Vøllestad et al., 1988). Indeed, when using the same duty cycle in the same muscle group, but at 40% MVC, the original group noted large reductions in phosphocreatine over the initial 30 min (Saugen et al., 1997).

**FIGURE 1:**
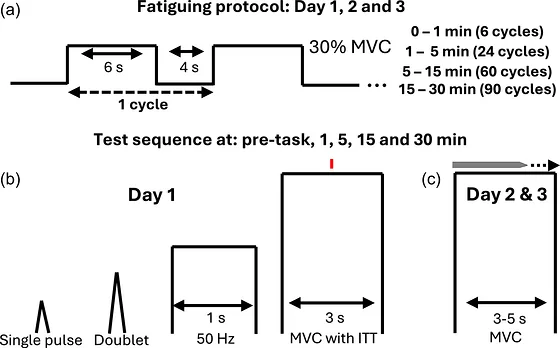
Schematic diagram of experimental protocol. (a) Schematic representation of the fatiguing protocol, which consisted of intermittent (duty cycle, 0.6 s) knee extensions at 30% MVC for 30 min. This protocol was repeated across 3 days. At each time point (pre‐task, 1, 5, 15 and 30 min) a test sequence was performed. (b) On day 1, the test sequence consisted of an electrically evoked twitch, doublet (100 Hz), 50 Hz (1 s) and the ITT protocol (i.e., MVC with superimposed doublet) with ∼2–3 s between contractions. (c) On days 2 and 3, the test sequence consisted of a 3–5 s knee‐extension MVC with intramuscular EMG recordings. Abbreviations: ITT, interpolated twitch technique; MVC, maximal voluntary contraction.

On the second and third day, the fatiguing protocol was repeated; however, the test sequence (see section 2.8 Days 2 and 3) was replaced with a brief 3–5 s MVC (Figure 1c). Furthermore, on these two days no electrical stimulation occurred, but instead, intramuscular electromyography (see section 2.5 Electromyography) was used to record MU trains during the MVCs (Figure 1c) and the submaximal (30% MVC) contractions.

### 2.7 Day 1: Experimental protocol

Using single pulses, maximal stimulation current was established as described above (see section 2.4 Electrical stimulation). Following ∼3 min rest, the interpolated twitch technique (ITT) was used to assess isometric voluntary activation (VA) (Todd et al., 2004). Briefly, a maximal doublet stimulus (100 Hz) was applied percutaneously over the quadriceps while at rest ∼2 s before, during the torque plateau of a 3 s knee‐extension MVC, and ∼2 s following the MVC with the muscle at rest. Following a 5 min rest period, the ITT protocol was repeated to ensure high VA (≥90%) during MVCs. A doublet was used to ensure a good signal‐to‐noise ratio (Hales & Gandevia, 1988).

Following a 5 min rest, the electrical current was adjusted such that 50 Hz stimulation (1 s) would evoke 50% of the isometric MVC torque. The current was increased in steps (∼5–15 mA), with a minimum of 30 s between contractions, until the torque target was reached. The 50% torque target was chosen to match the previous study using the same fatiguing protocol (Vøllestad et al., 1988). Furthermore, this torque output is tolerable for participants and is a reliable estimate of whole‐muscle contractile function in the quadriceps (Edwards et al., 1977).

After another 5 min of rest, a test sequence was performed. The sequence consisted of an electrically evoked twitch, doublet (100 Hz), 50 Hz (for 1 s) and the ITT protocol (i.e., MVC with superimposed doublet) with ∼2–3 s between contractions. An example of the test sequence is presented in Figure 2. After a further 5 min rest, the fatiguing protocol began (see section 2.6 Fatiguing protocol; Figure 1a).

**FIGURE 2:**
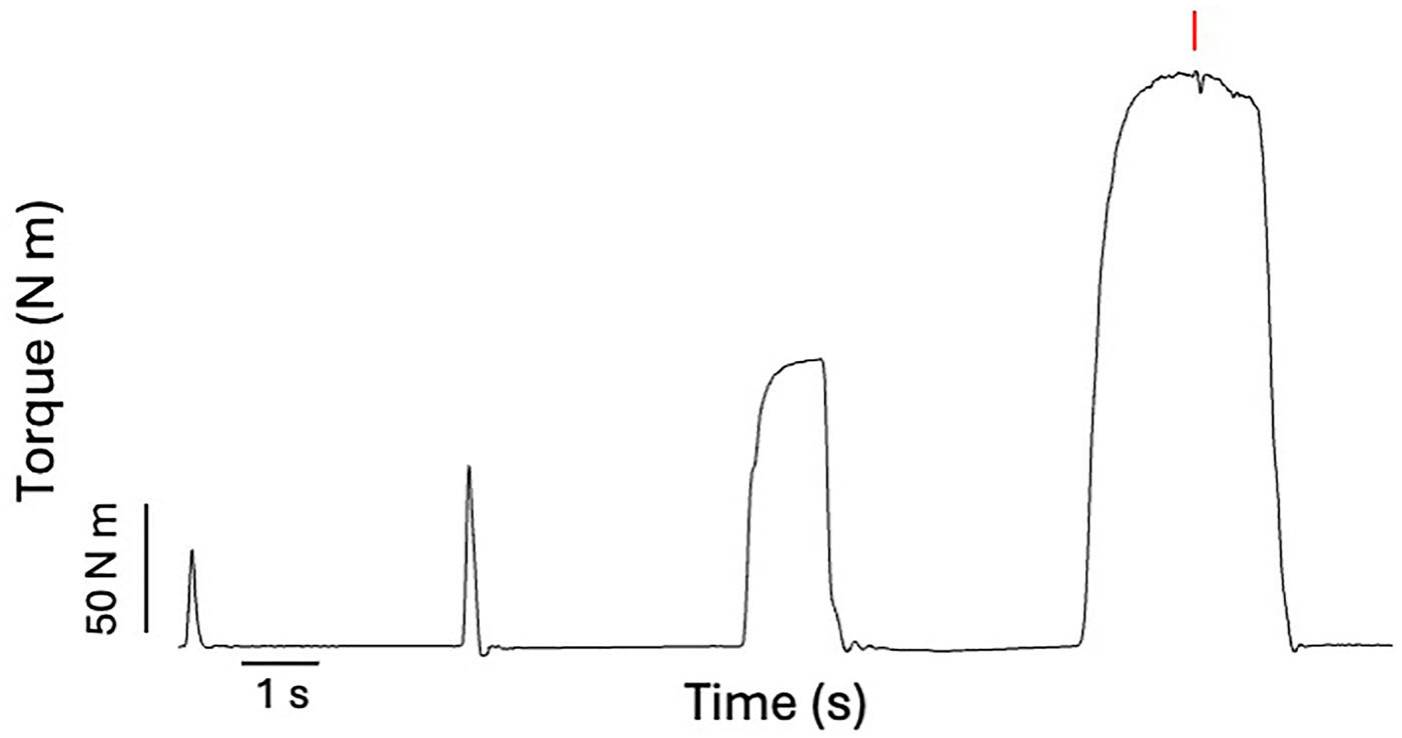
Exemplar torque traces from the test sequence performed on day 1. The sequence consisted of an electrically evoked twitch, doublet (100 Hz), 50 Hz (1 s) and the ITT protocol (i.e., MVC with superimposed doublet) with ∼2–3 s between contractions. The red vertical line indicates superimposed stimulation during MVC. Abbreviations: ITT, interpolated twitch technique; MVC, maximal voluntary contraction.

### 2.8 Days 2 and 3: Experimental protocol

Tungsten microelectrodes were inserted into the vastus lateralis and medialis as described above (see section 2.5). Participants produced three 3–5 s knee‐extension MVCs, separated by a minimum of 5 min rest. These assessments provided pre‐task (i.e., baseline) maximal MU firing rates.

After a 5 min rest, the fatiguing protocol used during day 1 was repeated (Figure 1a). At 1, 5, 15 and 30 min, a 3–5 s knee‐extension MVC was performed to record MU firing rates (Figure 1c).

### 2.9 Data analysis

All analysis was performed offline using Spike2 (Cambridge Electronic Design). Isometric MVC was defined as the mean torque value over a stable 1 s plateau of maximal voluntary efforts, and VA was assessed as previously described (Todd et al., 2004).

Electrically evoked twitch (single pulse), doublet (100 Hz) and 50 Hz peak torques were defined as the maximal values (in newton metres) obtained from each, respectively. For the twitch response, various measures of contractile rate were also assessed. The average twitch rate of torque development (RTD; in newton metres per millisecond) and rate of relaxation (RR; in newton metres per millisecond) were calculated as the mean slope from the initial deviation from baseline to peak torque, and the mean slope from peak torque to baseline, respectively. Normalized RTD (nRTD) and RR (nRR) were calculated as RTD (in newton metres per millisecond) and RR (in newton metres per millisecond) divided by peak torque (in newton metres) × 100, respectively, expressed as a percentage per millisecond. Twitch half‐relaxation time (HRT; in milliseconds) and contraction duration (time to peak torque + HRT; in milliseconds) were also calculated.

A comprehensive report of contractile rates is presented only for the twitch response. This was done to reduce redundancies and the volume of data presented, because the decline of half‐relaxation times during this protocol is not significantly different when assessed with a single pulse (i.e., twitch), 15 or 50 Hz stimulation (Vøllestad et al., 1997). Therefore, only a few crucial contractile rate assessments are reported here for the 100 Hz doublet and 50 Hz stimulation (1 s) to supplement the findings from the twitch response. For the doublet, nRTD, HRT and CT are reported, and they are analysed in a similar manner to the twitch response (described above). For the 50 Hz stimulation, peak RTD was calculated from the torque recording as the peak slope between the initial deviation from baseline and the peak torque over a moving 5 ms window. Normalized RTD was calculated as peak RTD (in newton metres per millisecond) divided by peak torque (in newton metres) ×100, expressed as a percentage per millisecond. Peak RTD was preferable because in response to 50 Hz stimulation there are two slopes, an initial steep slope followed by a shallow slope (Ruggiero et al., 2021). Therefore, calculating average RTD (described above) might be less informative and provide skewed values of little physiological relevance for the present question.

To assess muscle activity of the vastus lateralis, sEMG was used. Averaged root‐mean‐squared (RMS) amplitude was measured over a 2 s epoch during pre‐task knee‐extension MVCs. The RMS values (2 s epoch) during submaximal contractions at 30% MVC were normalized to the value obtained during the pre‐task MVC. Therefore, sEMG during submaximal contractions is presented as a percentage of maximal sEMG.

Analysis of single MUs was performed (Spike 2, Cambridge Electronic Design) as described previously (Connelly et al., 1999; Roos et al., 1999). Spike allocation was confirmed using a template‐matching algorithm and visual inspection by an experienced operator. The operator viewed sequential overlays of MU action potentials to confirm similar or progressively changing waveform shape and amplitude within an MU spike train. Additionally, to be included for firing rate analysis, a minimum of five contiguous MU action potentials per MU train and an interspike duration coefficient of variation ≤30% was required (Fuglevand, Winter et al., 1993). For submaximal contractions (30% MVC), firing rates were calculated from MUs in the vastus lateralis recorded from the three contractions prior to each test sequence (1, 5, 15 and 30 min) and were compared with the first three contractions of the task (representing pre‐task values).

### 2.10 Statistical analysis

All statistical analysis was performed in R (v.4.1.2). Linear mixed‐effects models with the *lme4* package (Bates et al., 2015) were used to determine whether the fixed effects of muscle group and time were significant predictors of maximal MU firing rate. Additionally, linear mixed‐effect models were used to assess whether the fixed effect of time was a significant predictor of submaximal MU firing rates. Each participant and their corresponding MUs were treated as random intercepts. The sEMG and VA were also compared using mixed linear models with the *lme4* package (Bates et al., 2015) to determine whether the fixed effect time was a significant predictor of RMS amplitude and muscle activation, respectively. Each participant was treated a random intercept. Using the lmerTest package (Kuznetsova et al., 2017), significance was calculated by comparing the full model (with effect of interest) against the null, which has the effect of interaction excluded. The lmerTest package uses Satterthwaite's method to approximate degrees of freedom and generate *P*‐values. *Post hoc* testing was done using the emmeans package (Lenth & Lenth, 2018), which examines the estimated marginal means with 95% confidence intervals. Tukey's method was used to adjust *P*‐values, and Kenward–Roger approximation was used to estimate degrees of freedom. Normality of data was tested with Shapiro–Wilk tests and visually with quantile–quantile plots. Separate one‐way repeated‐measures ANOVAs were used to compare the effect of time [(*F*_4,36_)], and data that were not normally distributed were compared using Friedman's test. *Post hoc* testing was performed using Student's paired *t*‐tests or Wilcoxon signed rank tests with Holm–Šídák corrections. Intraclass coefficients (ICCs) were used to evaluate test–retest reliability of the fatiguing tasks completed on separate days (Koo & Li, 2016). A Pearson correlation was used to measure a linear correlation between the submaximal sEMG and mean MU firing rates during the fatiguing task. Effect sizes were calculated with Cohen's *d*. For statistical comparisons, *P* ≤ 0.05 was considered significantly different.

## 3 RESULTS

### 3.1 Pre‐task

Electrically evoked twitch parameters at pre‐task (baseline) are presented in Table 1. Doublet (100 Hz) and 50 Hz (1 s) peak torque pre‐task values were 92.8 ± 29.5 and 150.5 ± 58.7 N m, respectively. Torque during pre‐task MVC was 291.1 ± 119.9 N m, and VA was 93.5% ± 4%.

**TABLE 1:** Pre‐task values of electrically evoked knee‐extensor twitch contractile properties.

Twitch parameter	Value
Peak torque, N m	52.5 ± 22
RTD, N m/ms	0.6 ± 0.26
nRTD, %/ms	1.2 ± 0.09
HRT, ms	76.7 ± 18
CT, ms	164.1 ± 20.4

Abbreviations: CT, contraction time; HRT, half‐relaxation time; nRTD, normalized rate of torque development; RTD, rate of torque development.

### 3.2 Fatiguing task: Contractile assessment, voluntary activation and electromyography

When intermittent assessments were made throughout the fatiguing task, there were significant effects of time (Figure 3; all *P* *<* 0.001) for electrically evoked peak torque measures of the twitch, doublet and 50 Hz, and at 30 min these measurements were 59.9% ± 28.9% (*d* = 1.17), 68.3% ± 25% (*d* = 1.13) and 50.7% ± 17% (*d* = 1.91) of pre‐task values, respectively (all *P* ≤ 0.02).

**FIGURE 3:**
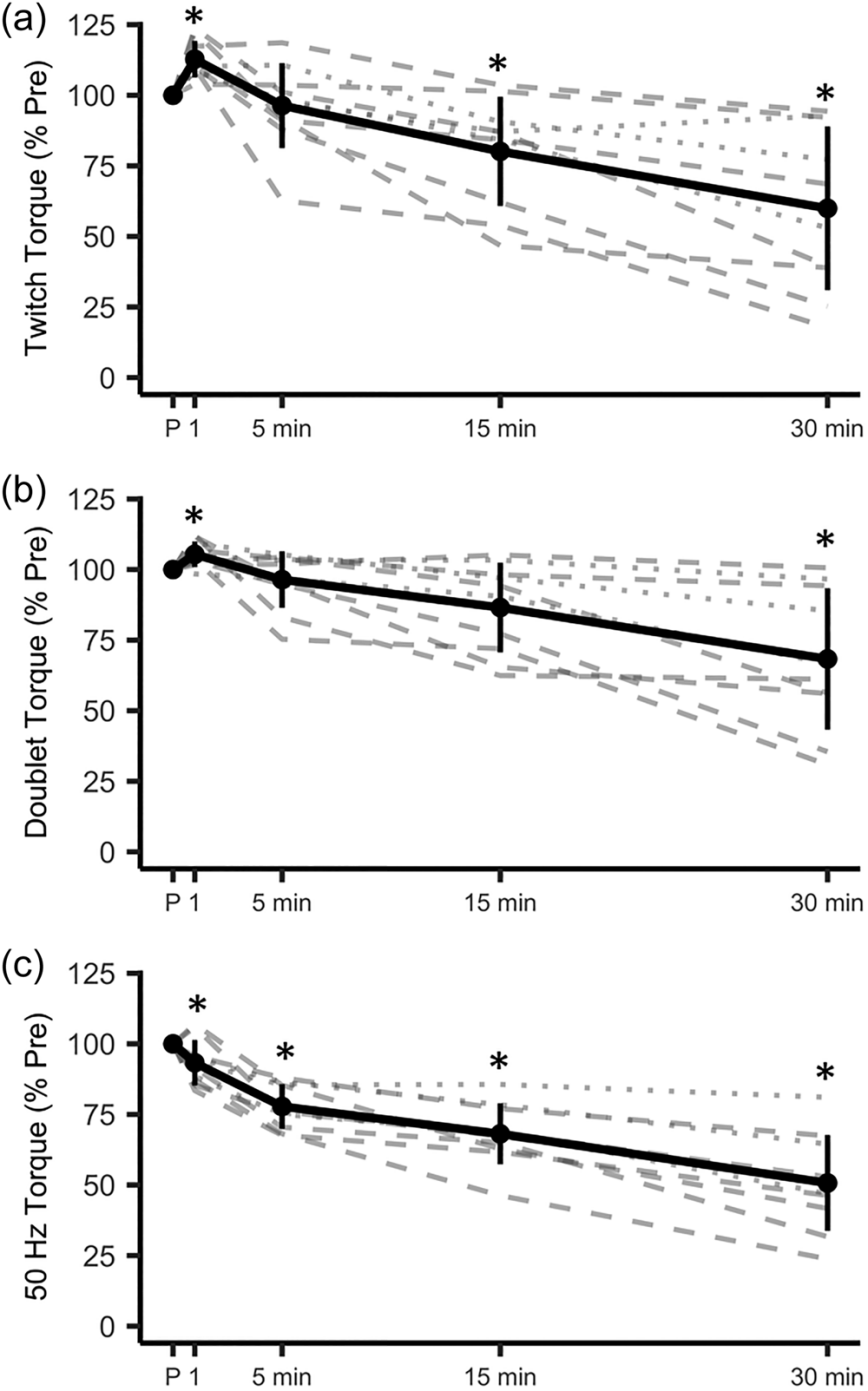
Twitch (a), doublet (100 Hz; b) and 50 Hz torque (c) during the fatiguing task. Values are expressed as a percentage of pre‐task (i.e., baseline) values denoted as black circles and presented as the mean ± SD. Dashed and dotted lines represent data from individual male and female participants, respectively. *Significant difference from pre‐task (denoted as P). *n* = 10.

There was a significant effect of time for twitch RTD and nRTD (both *P* < 0.001; Figure 4a, b). At 30 min, RTD was 65.2% ± 10.1% of pre‐task values (*P* = 0.02, *d* = 0.99); conversely, nRTD was 114.5% ± 13.7% of pre‐task values (*P* = 0.03, *d* = 1.1).

**FIGURE 4:**
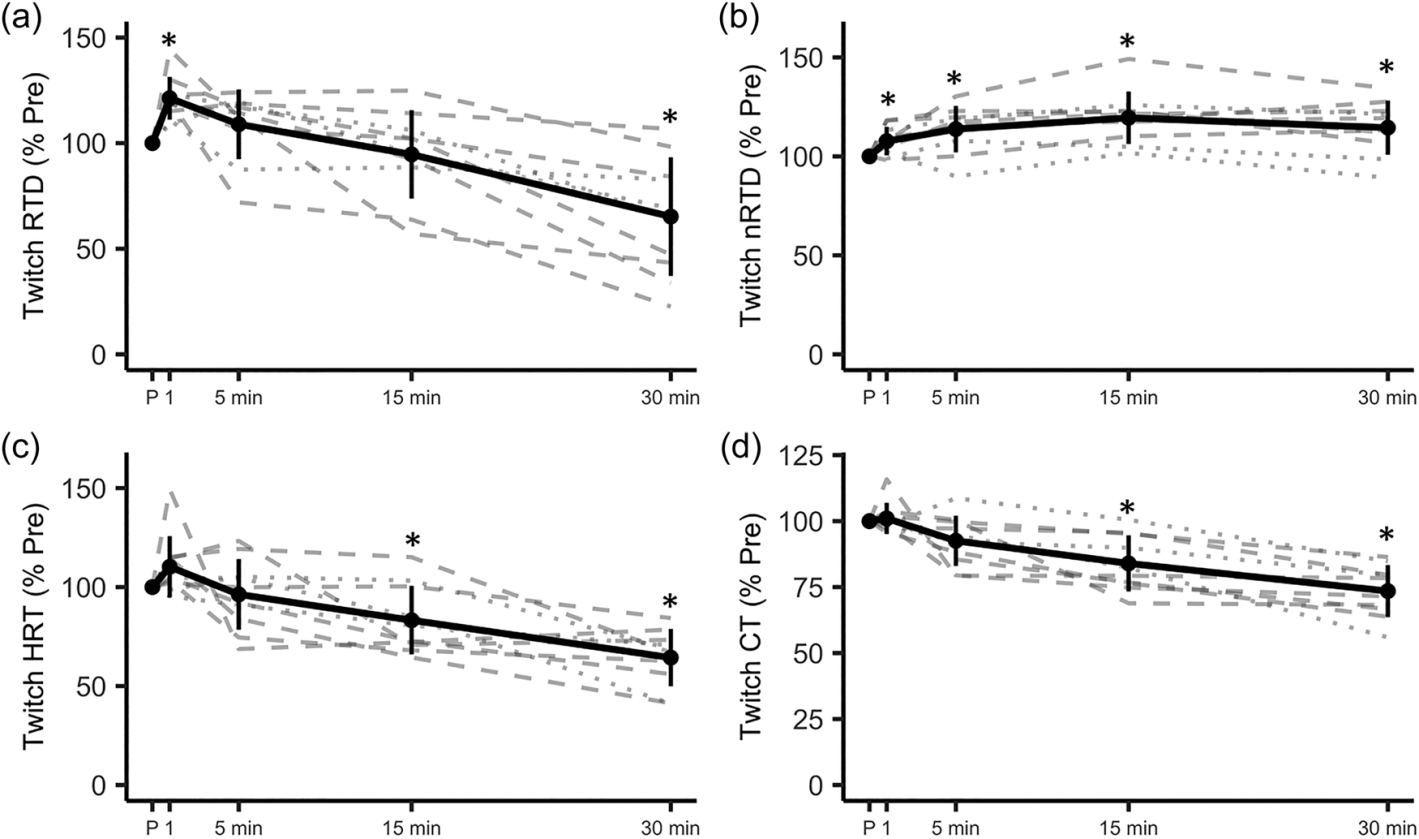
Twitch contractile rates during the fatiguing task. (a) Twitch RTD. (b) nRTD. (c) HRT. (d) CT. Values are expressed as a percentage of pre‐task (i.e., baseline) values denoted as black circles and presented as the mean ± SD. Dashed and dotted lines represent data from individual male and female participants, respectively. *Significant difference from pre‐task (denoted as P). *n* = 10. Abbreviations: CT, contraction time; HRT, half‐relaxation time; nRTD, normalized rate of torque development; RTD, rate of torque development.

There was a significant effect of time for twitch HRT and CT (both *P* < 0.001; Figure 4c, d). At 30 min, these measurements were 64.4% ± 14.5% and 73.5% ± 9.8% of pre‐task values, respectively (both *P* < 0.001, *d* = 2 and 1.8, respectively).

There was no significant effect of time for twitch RR (*P* = 0.91), and the range of mean values at all time points was 96.8%–103.4% relative to pre‐task (−0.34 ± 0.13 N m/ms). However, there was a significant effect of time for nRR (*P* < 0.001), and at 30 min it was 163.8% ± 44.2% of pre‐task values (−0.69%/ms ± 0.1%/ms, *P* < 0.001, *d* = 1.38). However, there were no significant differences at 1 (91.9% ± 10.8%, *P* = 0.07), 5 (107% ± 20.7%, *P* = 0.54) and 15 min (124.2% ± 23.3%, *P* = 0.08) compared with pre‐task values.

For the doublet response, there were significant effects of time for nRTD (*P *< 0.001), HRT (*P *= 0.002) and CT (*P *< 0.001). At 30 min, these values were 130.3% ± 27% (*d* = 1.35), 73.6% ± 22.5% (*d* = 0.96) and 74.9% ± 13.5% (all *P* ≤ 0.04, *d* = 1.94) of pre‐task values (1.03%/ms ± 0.18%/ms, 73.6 ± 22.5 ms and 175.6 ± 13.7 ms, respectively). For the 50 Hz response, there was a significant effect of time for nRTD (*P *< 0.001) and HRT (*P* = 0.001), and at 30 min these were 126.4% ± 16.4% and 84.5% ± 15.4% (both *P* < 0.001, *d* = 0.3 and 0.75, respectively) of pre‐task values (0.75%/ms ± 0.15%/ms and 105.4 ± 31.4 ms, respectively).

There was a significant effect of time for MVC torque (*P* < 0.001), VA (*P* = 0.001) and sEMG during MVCs (*P* < 0.001; Figure 5). At 30 min, MVC torque and sEMG amplitude were 61.7% ± 17.3% and 75.74% ± 14.1% of pre‐task values, respectively (*d* = 1.73 and 1.94, respectively, both *P* < 0.001). At 30 min, there was a significant decline in VA from 93.5% ± 4% to 88% ± 7.9% (*P* = 0.01, *d *= 0.68).

**FIGURE 5:**
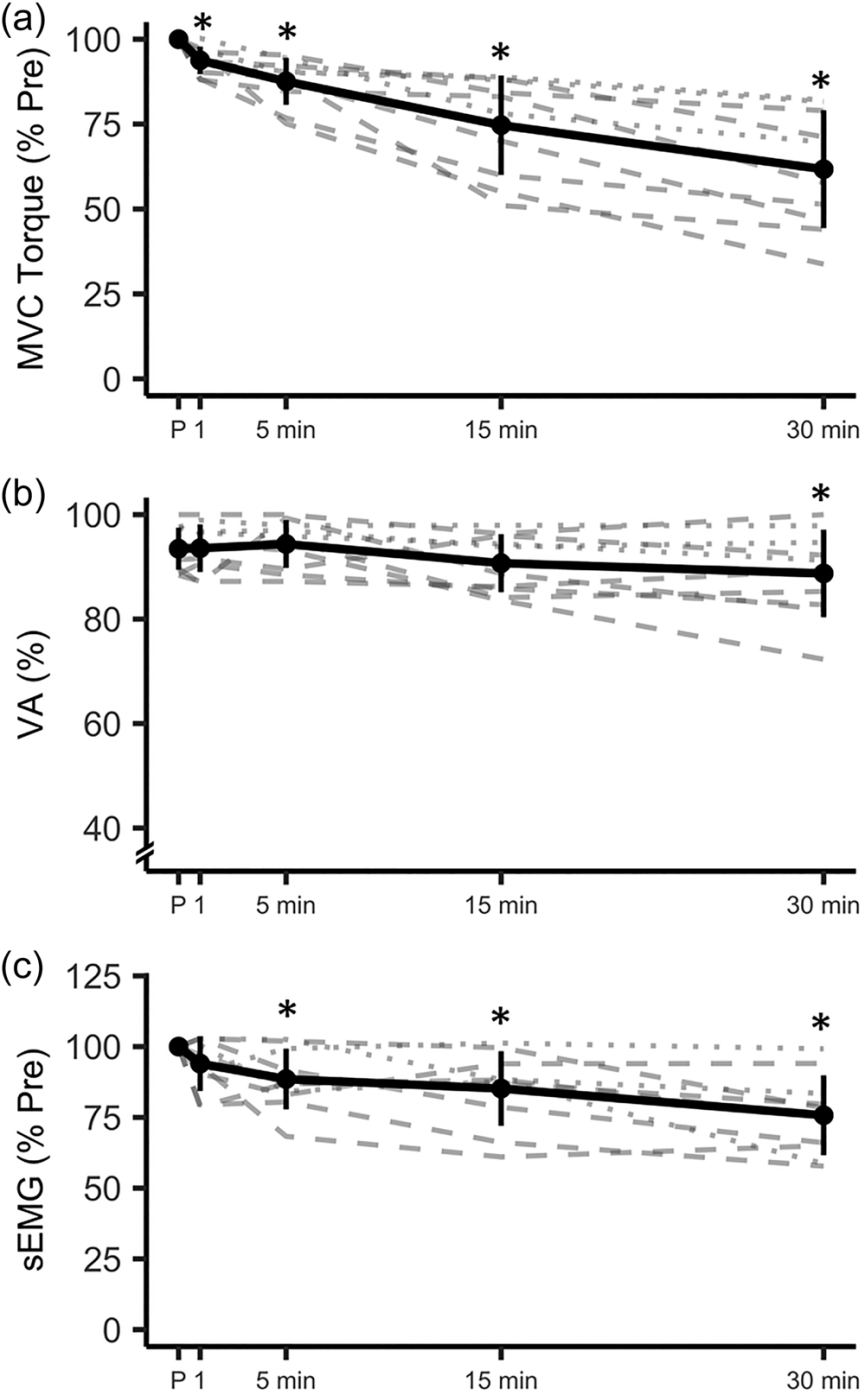
Torque during MVC (a), VA (b) and maximal sEMG (c) during the fatiguing task. Values are expressed as a percentage of pre‐task (i.e., baseline) values denoted as black circles and presented as the mean ± SD. Dashed and dotted lines represent data from individual male and female participants, respectively. *Significant difference from pre‐task (denoted as P). *n* = 10. Abbreviations: MVC, maximal voluntary contraction; sEMG, surface electromyography; VA, voluntary activation.

A summary of MU characteristics recorded during 30% MVCs is presented in Table 2. Exemplar MU recordings are shown in Figure 6. There was an effect of time for mean MU firing rates (*P *< 0.001) and sEMG (*P* < 0.001) assessed during the 30% MVCs throughout the fatiguing task (Figure 7). At 30 min, firing rates increased by 16% and sEMG increased from 27.5% ± 9.7% to 48.2% ± 14.6% of maximum (*P* < 0.001). There was a modest linear relationship between the increase in mean firing rates and sEMG throughout the fatiguing task (*r* = 0.36, *P* = 0.01).

**TABLE 2:** Motor unit characteristics from the vastus lateralis (*n* = 605) recorded during submaximal efforts at 30% maximal voluntary contraction during the 30 min fatiguing task.

Parameter	Pre‐task	1 min	5 min	15 min	30 min
No. of MU trains	93	96	137	134	145
No. of MU trains per participant	9.3 ± 3.2	9.6 ± 4.4	13.7 ± 4.7	13.4 ± 3.1	14.5 ± 5.5
No. of ISIs per MU train	7.2 ± 3.9	6.6 ± 2.8	7.4 ± 4.8	7.7 ± 5.8	7.7 ± 4.4
CoV (%)	8.6 ± 5.2	10.4 ± 6.1	8.6 ± 4.1	9.7 ± 5.4	9.6 ± 4.6

*Note*: Firing rates are presented in Figure 7.

Abbreviations: CoV, coefficient of variation (percentage); ISI, interspike interval; MU, motor unit.

**FIGURE 6:**
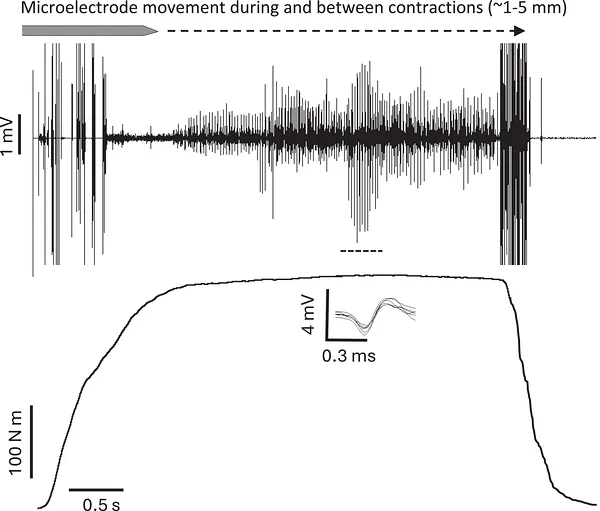
Exemplar motor unit recording during a pre‐task (i.e., baseline) maximal voluntary contraction. Top panel, schematic diagram of tungsten microelectrode movement during an active contraction. Middle panel, intramuscular EMG recording from a single tungsten microelectrode inserted into the vastus lateralis. Bottom panel, torque trace during maximal voluntary contraction, with an overlay of action potentials of a single motor unit highlighted from the middle panel.

**FIGURE 7:**
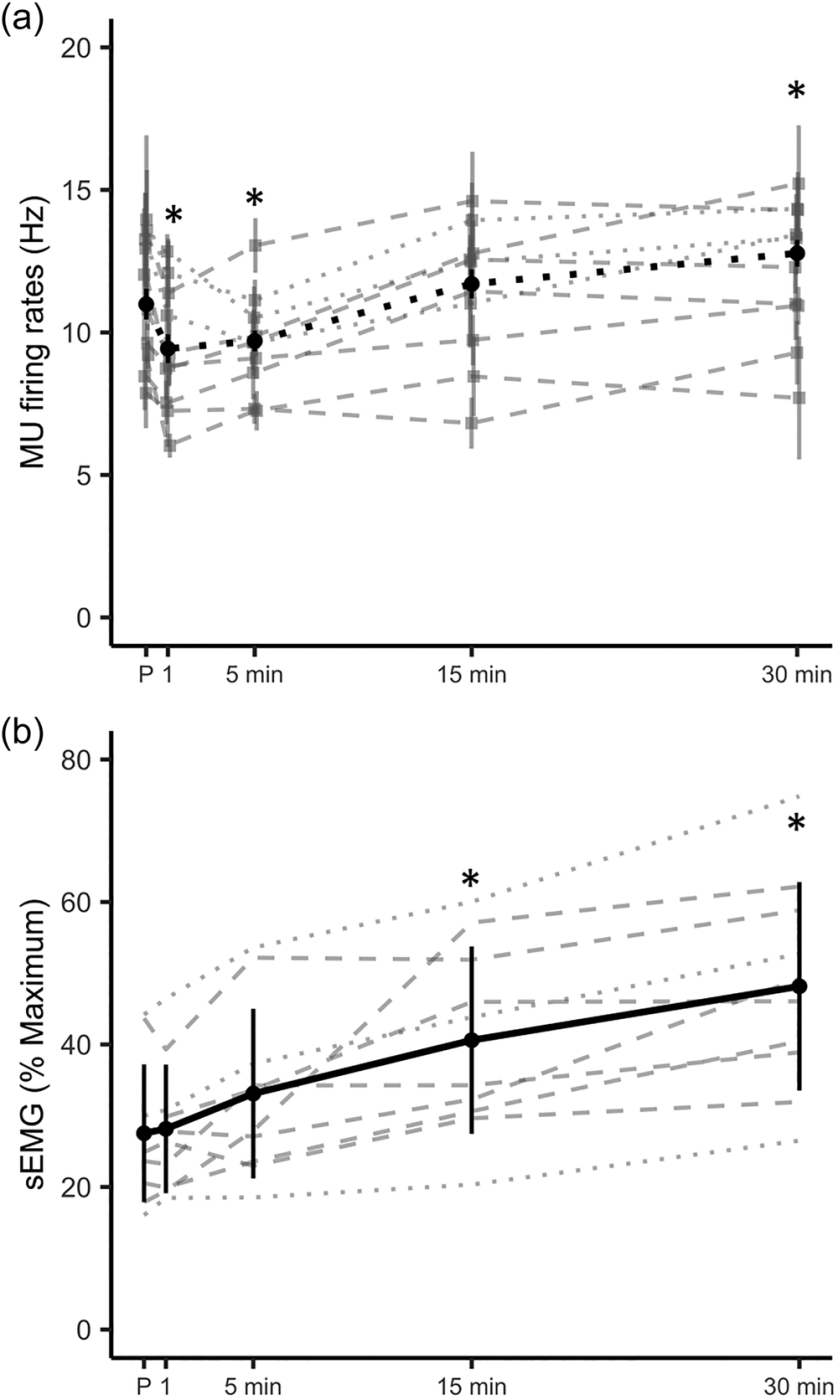
Submaximal (i.e., 30% MVC) motor unit firing rates (a) and sEMG (b) during the fatiguing task. Motor unit firing rates are expressed in hertz, and sEMG is a percentage of maximum (i.e., relative to pre‐task MVC). Values are denoted as black circles and presented as the mean ± 95% confidence interval for firing rates and ± SD for sEMG. (a) Each transparent grey square represents the mean firing rate of an individual, and dashed and dotted lines represent males and females, respectively. Error bars are 95% confidence intervals. (b) Dashed and dotted lines represent data from individual male and female participants, respectively. These data were collected on days 2 and 3 (see Figure 1). *Significant difference from pre‐task (denoted as P). *n* = 10; total motor units in (a) = 605. Abbreviations: MVC, maximal voluntary contraction; sEMG, surface electromyography.

The fatiguing task was repeated across three days to ensure a substantive MU sample (see section 2 MATERIALS AND METHODS). Across days, MVC torque loss demonstrated good reliability (ICC = 0.8, *P* = 0.002), and the decline at 30 min was not significantly different across days (*P* = 0.17).

### 3.3 Fatiguing task: Maximal MU firing rates

A summary of MU characteristics recorded during MVCs is presented in Table 3. Exemplar MU recordings are shown in Figure 6. There was no significant effect of muscle (vastus lateralis and medialis) or a muscle × time interaction (*P* = 0.18 and *P* = 0.17, respectively) on MU firing rates assessed intermittently during MVCs throughout the 30 min fatiguing task. Therefore, MUs from both muscles were combined to represent the quadriceps muscle group better (pre‐task firing rate of vastus lateralis and medialis were 24.7 ± 8.8 and 24.8 ± 10.2 Hz, respectively). When combined, there was no significant effect of time on maximal MU firing rates (χ^2^(4) = 6.5, *P* = 0.16; Figure 8).

**TABLE 3:** Motor unit characteristics from the vastus lateralis (*n* = 646) and medialis (*n* = 306) muscles recorded during maximal voluntary contractions intermittently performed during the 30 min fatiguing task.

Parameter	Pre‐task		1 min		5 min		15 min		30 min	
	VL	VM	VL	VM	VL	VM	VL	VM	VL	VM
No. of MU trains	269	100	50	28	75	53	90	51	162	74
No. of MU trains per participant	26. 9 ± 10.9	10 ± 4.8	5 ± 2.4	2.8 ± 1.2	7.5 ± 3.1	5.3 ± 1.4	9 ± 3	5.1 ± 2.2	16.2 ± 4.2	7.4 ± 2.8
No. of ISIs per MU train	6 ± 2.7	6.1 ± 3.1	6.1 ± 2.6	6.4 ± 3	6.6 ± 3.5	6.6 ± 6.1	6.1 ± 3.4	6.4 ± 2.4	6 ± 2.9	6.8 ± 3.2
CoV (%)	12.1 ± 5.7	13.1 ± 5.7	14.2 ± 7.4	13.6 ± 6.8	12.9 ± 6.5	13.2 ± 5.9	13.6 ± 6.6	13.1 ± 6.6	11.9 ± 5.9	13.1 ± 5.9

*Note*: Firing rates are presented in Figure 8.

Abbreviations: CoV, coefficient of variation (percentage); ISI, interspike interval; MU, motor unit.

**FIGURE 8:**
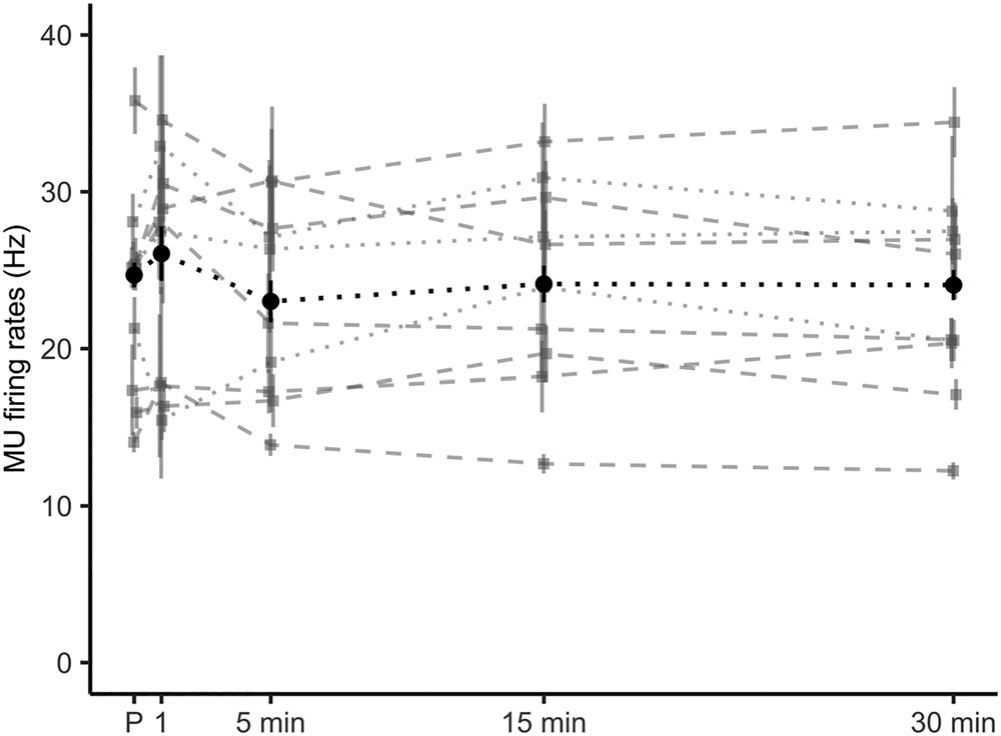
Maximal motor unit firing rates during the fatiguing tasks. The black circles represent the ensemble mean of all participants. Each transparent grey square represents the mean firing rate of an individual, and dashed and dotted lines represent males and females, respectively. Error bars for all conditions are 95% confidence intervals. These data were collected on days 2 and 3 (see Figure 1). Values are expressed in hertz. *Significant difference from pre‐task (denoted as P). *n* = 10, total motor units = 952.

## 4 DISCUSSION

Despite large reductions in MVC torque and increases in evoked contractile speed during the submaximal fatiguing task, maximal MU firing rates were unaffected. Therefore, these results indicate that reductions in maximal torque generation, contractile slowing and decreased maximal MU firing rates are not invariable correlates of muscle fatigue. Importantly, these results indicate that reduced MVC torque can be dissociated from the usual slowing of both maximal firing rates and contractile speed when muscle fatigue is induced with submaximal contractions that are likely to have negligible metabolic disturbances. Overall, the constancy of maximal firing rates despite large reductions in maximal torque generation supports that rate moderation usually reported with high‐intensity fatiguing activation may be attributable to reflexive inhibition from changes in muscle metabolites. The submaximal task used here has strengthened that premise.

Reflexive inhibition of maximal MU firing rates during fatiguing exercise is not a new concept (Bigland‐Ritchie et al., 1986; Gandevia et al., 1990; Macefield et al., 1993). However, the specific feedback involved and to what extent these factors moderate maximal firing rates has remained unclear (Gandevia, 1993; Nordstrom et al., 2007; Stuart & Callister, 1993). We aimed to test the hypothesis that accumulation of muscle metabolites is the primary factor involved for peripheral firing rate regulation with fatigue (Bigland‐Ritchie et al., 1986). To that end, we mimicked closely an intermittent submaximal fatiguing protocol that has been previously shown to induce negligible metabolic disturbances (Vøllestad et al., 1988) despite substantial reductions in MVC torque (∼40%, which was well matched in our experiments, see Figure 5a). Specifically, in the prior study intramuscular concentrations of glycogen, lactate, creatine phosphate and ATP did not change significantly (Vøllestad et al., 1988). Following this same protocol, we found that maximal MU firing rates remained unaffected (Figure 8), probably because large metabolic disturbances did not occur throughout the fatiguing task (Vøllestad et al., 1988). Thus, our results indicate that afferent feedback, particularly from mechano‐ and chemosensitive group III/IV fibres, might be the primary mechanism for the decline in maximal MU firing rates during fatiguing activation.

Contractile slowing is a common feature associated with fatiguing exercise, which is generally attributed to large alterations in concentrations of high‐energy substrates and metabolites (Cady, Elshove et al., 1989; Dawson et al., 1980; Edwards et al., 1975). In contrast, the fatiguing protocol used here increased various measures of contractile speed, despite torque loss, again probably because of negligible metabolic disturbances (Vøllestad et al., 1988); however, the mechanisms underlying this observation are not entirely clear. Thus, the ability to disassociate large reductions in torque from contractile slowing by using a submaximal task has provided new support for this concept and indicates that depression of maximal firing rates is not a universal feature of muscle fatigue.

Muscle temperature has been shown to increase by ∼3°C during a similar submaximal (30%) intermittent fatiguing protocol (Saugen & Vøllestad, 1996), and it is established that warmer temperatures increase contractile speed (Lännergren & Westerblad, 1987), which could explain the faster contractile rates. Conversely, increases in contractile speed could be attributable to modifications in muscle fibre activation throughout the fatiguing task. To produce submaximal contractions, only a fraction of the MU pool is required (Heckman & Enoka, 2012). As the fatiguing task progresses, earlier recruited MUs will begin to fatigue and contribute less to torque in response to whole‐muscle stimulation. Therefore, in response to electrical stimulation, the later‐recruited (larger) MUs would produce a greater fraction of the whole‐muscle output, especially if they are not yet recruited during the fatiguing task (Vøllestad et al., 1997). Thus, whole‐muscle contractile speed could increase as the torque response to stimulation has a greater influence from larger MUs (Edman, 2014) (which might be composed of more fast‐type fibres) (Burke, 1981). Although the exact mechanism(s) for the observed increase in contractile speed during this fatiguing protocol requires further exploration and confirmation, importantly for the current experiment the contractile speed did not slow.

As torque‐generating capacity declines, the relative intensity of the pre‐set fatiguing task target increases. In response, greater muscle activation is required to compensate and meet the task demand. Accordingly, by 30 min we observed a small increase in MU firing rates (16%), following an initial minor reduction (Martinez‐Valdes et al., 2020), but a large increase in sEMG (75%) when assessed during the submaximal 30% MVCs (Figure 7). Recruitment was not assessed specifically, but the modest changes in firing rate are unlikely to explain fully the large increase in sEMG, and recruitment of previously inactive MUs is likely to have occurred (Garland et al., 1994, 1997). Indeed, there was only a moderate relationship (*r* = 0.36) between the increases in firing rates and sEMG decline. The mean firing rate range of the vastus lateralis and medialis is relatively modest (∼8–26 Hz; Fanous et al., 2026a, 2026b; Jakobi & Cafarelli, 1998; Rich & Cafarelli, 2000; Roos et al., 1999) compared with other limb muscles, and thus recruitment might have been favoured throughout the task. Collectively, these findings indicate that as exercise progresses a larger proportion of the MU pool contributes to torque in this submaximal fatiguing task. However, because participants were required to maintain a relative torque, rather than activation level (e.g., sEMG), participants were likely to be using different amounts of their motor pool to maintain the task demand.

During acute (e.g., <60 s) MVCs, muscle contractile speed slows, in addition to the decline in both maximal MU firing rate and torque (Bigland‐Ritchie et al., 1983a, 1986; Bigland‐Ritchie & Woods, 1984; Zero et al., 2025). Indeed, there is evidence to suggest that concurrent slowing of maximal firing rates and contractile speed might be beneficial for torque preservation during high‐intensity isometric contractions (i.e., the muscle wisdom hypothesis). However, our findings indicate that despite the large reduction (∼40%) in maximal torque generation, contractile speed can vary independently from the unchanging maximal firing rates. The muscle wisdom hypothesis suggests that during a sustained high‐intensity (e.g., >75% maximum) isometric contraction, the lowered firing rates provide adequate excitation in part because it matches the fatigue‐induced contractile slowing (Bigland‐Ritchie et al., 1979; Jones et al., 1979; Marsden et al., 1983; Zero & Rice, 2025). Despite the antithetical findings of a faster contractile speed and no change in maximal firing rates, our results can be interpreted as complementary to the hypothesis. From this perspective, maximal firing rates did not decline because it was not beneficial, given that contractile speed did not slow. Rather, because contractile speed was faster, an increase in maximal firing rates might have been beneficial for torque preservation. If true, this could be interpreted in multiple ways.

First, there is a ceiling effect of maximal MU firing rates, above which no rate coding can occur to compensate for the faster contractile rates. Conversely, it could be argued that MUs have an intrinsic capability to increase their rates, but in this case they do not. For example, MUs can fire at >100 Hz for a few intervals (e.g., doublets) (Desmedt & Godaux, 1977; Van Cutsem et al., 1998) and mean firing rates during maximal concentric contractions (i.e., joint angle reduction) are reported to be ∼60% higher than isometric MVCs (Harwood et al., 2011). Therefore, MUs are intrinsically capable of discharging at higher rates, and the inability to rate code during isometric MVCs could be viewed as a central output limitation in this task. This maximal firing rate might be sufficient in the pre‐task (baseline) state, but when contractile speed increases this rate might no longer be adequate, hence input becomes suboptimal despite the maintained firing rate. To compensate for the combination of increased contractile speed and decreased torque generation, mean firing rates during submaximal contractions increased by the end of the task (Figure 7). Therefore, the ability of MUs to adapt and rate code is constrained by the task demand. Despite the interpretation of either a ceiling effect or a central limitation, the inability of MUs to increase their maximal firing rate to match the faster contractile speed was a likely contributor to torque loss.

Throughout the fatiguing task, maximal sEMG declined, and at 30 min the VA was impaired by ∼6%. Despite the inherent limitations of interpreting sEMG (Farina et al., 2004), it remains reasonable to infer that MU decruitment is likely to have contributed to the observed reduction in maximal sEMG amplitude, as supported by the concurrent decrease in VA at 30 min and the absence of change in maximal MU firing rates. However, there is a large difference (∼6% and 23%) in the magnitude of decline between these two measurements (Figure 5), which could be attributable to a combination of their respective limitations. The sEMG signal is most influenced by superficial MUs (Fuglevand, Winter et al., 1993; Merletti et al., 2008), and in the vastus lateralis larger MUs are more prominent superficially (Knight & Kamen, 2005), in addition to a greater distribution of type II fibres (Lexell et al., 1983). Simulation data sets have shown that removal of the 60 smallest MUs caused only a ∼6% reduction in the surface‐detected M‐wave response; however, removal of the eight largest caused a ∼25% reduction (Keenan et al. 2006). This is particularly important because firing rate cessation or decruitment might be most apparent in higher‐threshold (larger) MUs (Grimby et al., 1981; Marsden et al., 1983). Therefore, any firing rate cessation of large or superficial MUs would substantially reduce the sEMG signal. Additionally, in the present study muscle belly stimulation was preferred, given that electrode placement in the femoral canal can be displaced easily as the activated muscles contract during trains of stimuli, leading to inconsistent torque measurements (see Yacyshyn & McNeil, 2022). However, even with maximal stimulation over the muscle belly, the entire quadriceps MU pool is not excited, hence the sensitivity of the VA measure might be affected (Osborne et al., 2023). Therefore, the observed reduction in VA could have been underestimated. Reductions in VA are reported following prolonged (e.g., >10 min) submaximal contractions, which could be attributable to increased corticospinal inhibition (Henderson et al., 2022; McNeil et al., 2011; Søgaard et al., 2006), reduced motoneuronal excitability (McNeil et al., 2011) or depletion of serotonergic neuromodulation (Fornal et al., 2006).

Large metabolic disturbances in the peripheral muscle are well‐established mechanisms contributing to muscle fatigue (torque loss following contractile activation). However, in response to the submaximal fatiguing protocol, electrically evoked and MVC torque loss occurred despite previously reported negligible metabolic disturbances (Vøllestad et al., 1988). The minor reduction in VA and the lack of change in firing rates during isometric MVCs (as discussed above) could contribute to reductions in MVC torque. However, electrically evoked torque loss was probably attributable to impairments in excitation–contraction coupling. Impaired action potential propagation can occur during sustained submaximal activation (Fuglevand, Zackowski et al., 1993), which could lead to inadequate engagement of the sarcolemma voltage sensors or t‐tubules. This might also explain the reduction in the submaximal sEMG response independent of changes in MU recruitment or firing rate. Furthermore, extended elevations of [Ca^2+^]_i_, as expected during a prolonged fatiguing task, lead to a reduction in Ca^2+^ release, even in the absence of large metabolic changes (Chin & Allen, 1996). In addition, sustained levels of Ca^2+^ will activate calpains, which initiate breakdown of structural proteins, leading to degradation of key proteins involved in excitation–contraction coupling (Lamb, 2009; Li et al., 2016; Murphy, 2010; Place et al., 2015). Although specific mechanisms cannot be established with the present experiments, it appears that the primary mechanism for torque loss in this task was impairments in various steps of excitation–contraction coupling (Vøllestad et al., 1988).

### 4.1 Considerations

In response to fatiguing stimulation, changes in contractile rates of single MUs vary (Thomas et al., 1991), and this variance might be dependent on the MU type, at least in the cat (Bigland‐Ritchie et al., 1998; Dubose et al., 1987). In response to whole‐muscle stimulation, the mechanical output is a summated response of all the activated MUs. Therefore, individual differences in single MU contractility are not captured, but they would contribute to the whole‐muscle contractile rates. Our technique to record MUs relied on large population sampling from active small and large manipulations of the intramuscular electrode, and our averaged rates align with previous findings in these muscles (Fanous et al., 2026a, 2026b; Jakobi & Cafarelli, 1998; Rich & Cafarelli, 2000; Roos et al., 1999). With this approach, specific MU types cannot be determined, and individual MUs were not followed throughout the protocol. If there was potential firing rate moderation during MVCs within a specific MU type, it was too small to affect the overall population mean.

Despite a continued increase in muscle activation during the submaximal fatiguing task, evidenced by an increase in sEMG and submaximal MU firing rates by 30 min (Figure 7), there is a likelihood that some MUs were not active or were not required. Therefore, it might be argued that it is not surprising that mean firing rates derived from population sampling during MVCs were not reduced. However, there are several lines of evidence to support that the inhibitory effect of group III/IV afferent feedback is widespread in humans during MVCs (Kennedy et al., 2013, 2014). For example, voluntary drive is reduced (assessed with superimposed doublets) in a non‐fatigued antagonist (Kennedy et al., 2013) and a proximal ipsilateral muscle (Finn et al., 2020; Kennedy et al., 2014) when a cuff is used to maintain ischaemia and prolong stimulation of group III/IV afferents after fatiguing contractions. Therefore, if group III/IV afferents were highly stimulated in response to MU activation in our fatiguing task, we should have widespread inhibitory effects on the quadriceps MU pool. There would probably be some minor group III/IV afferent activation even at the low contraction intensities of the fatiguing protocol (Adreani et al., 1997); however, this was presumably not large enough to induce any detectable changes in MU firing rate. Furthermore, we would expect a ∼50% reduction in maximal MU firing rates in response to a ∼40% MVC torque loss induced from sustained high‐intensity contractions when group III/IV afferents are highly stimulated (Bigland‐Ritchie et al., 1983b, 1986; Zero et al., 2023). In support, we observed no reduction in maximal firing rates throughout the submaximal fatiguing task (Figure 8).

Work using a combination of post‐contraction blood‐flow occlusion and MU recording strongly supports the concept that prolonged activation of group III/IV afferents reduces maximal firing rates (Bigland‐Ritchie et al., 1986; Woods et al., 1987). However, when a similar post‐contraction blood‐flow occlusion approach is applied, stimulated measures of motoneuron excitability (cervicomedullary motor evoked potential) are not affected in the biceps brachii (Butler et al., 2003), and this concept has been supported recently using lumbar intrathecal fentanyl to attenuate group III/IV feedback (Laginestra et al., 2026). The discrepancies among studies might reflect the distinct differences in which the motoneuron is activated. For example, voluntary contractions induce repetitive spiking in response to non‐steady‐state input (Heckman & Enoka, 2012) compared with the artificially induced brief, but intense stimuli required for cervicomedullary motor evoked potential assessment (Taylor, 2006). When considered collectively, these findings could be interpreted that motoneuron excitability is not affected by group III/IV activation, but that voluntary firing rates might be lower because of suboptimal descending drive onto the motoneuron pool despite increased cortical excitation, which probably does not compensate for the marked spinal depression (Gandevia et al., 1996; Laginestra et al., 2026). Therefore, there is no meaningful discrepancy between these studies; although the primary outcomes are related, they are not synonymous measures.

Our results are supported by a previous report that assessed maximal MU firing rates after a submaximal task (Thomas & Del Valle, 2001). That protocol consisted of 6 s contractions at 50% of MVC, followed by 4 s of rest, which was repeated until MVC torque (assessed every 5 min) was reduced by 50%. They indicated that despite the intermittent and submaximal nature of the task, large metabolic disturbances would probably have occurred, and indeed by task completion maximal MU firing rates declined by ∼30%. Thus, our findings of no significant change to maximal firing rates are not a result of the use of an intermittent submaximal task per se, but rather that our task induced presumably negligible metabolic disturbances (Vøllestad et al., 1988). Overall, our findings support a prominent role of reflexive inhibition from changes in muscle metabolites that reduce maximal MU firing rates. However, group III/IV muscle afferents are polymodal and are also sensitive to mechanical distortion, temperature and noxious stimuli, and these factors might also regulate maximal MU firing rates in part (Garland & Kaufman, 1995). The underlying mechanisms altering firing rate behaviour are task dependent, and reflexive inhibition might not be as influential in moderating firing rates during submaximal contraction intensities, in comparison to other factors that have been described elsewhere (Garland & Kaufman, 1995; Griffin et al., 2001; Nordstrom et al., 2007).

## 5 CONCLUSION

In response to fatiguing activation, contractile speed, maximal torque generation and maximal MU firing rate can vary independently. A reduction in maximal MU firing rates is not an invariable consequence of reductions in maximal torque generation, nor are increases in contractile speed. During fatiguing intermittent submaximal contractions, MUs rate code to compensate for reduced torque generation and faster contractile speeds. Conversely, during maximal contraction the firing rates cannot increase to compensate for these peripheral adaptations. Thus, the ability for MUs to rate code is constrained to the task demand. By using a fatiguing task, in which it is presumed that negligible metabolic disturbances occur (Vøllestad et al., 1988), the constancy of maximal firing rates despite large reductions in maximal torque generation strongly supports the original findings and speculations (Bigland‐Ritchie et al., 1986) that maximal rate reductions with fatiguing activation are likely to be attributable to reflexive inhibition from changes in muscle metabolites.

## AUTHOR CONTRIBUTIONS

Alexander M. Zero, Raaj A. Dudani, Jacob Fanous and Charles L. Rice contributed to conception or design of the work, acquisition, analysis or interpretation of data for the work and drafting the work or revising it critically for intellectual content. All authors approved the final version of the manuscript and agree to be accountable for all aspects of the work in ensuring that questions related to the accuracy or integrity of any part of the work are appropriately investigated and resolved. All persons designated as authors qualify for authorship, and all those who qualify for authorship are listed.

## CONFLICT OF INTEREST

The authors declare no conflict of interest.

## GENERATIVE AI STATEMENT

No generative artificial intelligence tools were used in the preparation of this work.

## Data Availability

Data are available upon reasonable request.
